# *Trypanosoma* (*Megatrypanum*) *lainsoni* n. sp. from *Mesomys hispidus* (Rodentia: Echimyidae) in Brazil: trypomastigotes described from experimentally infected laboratory mice

**DOI:** 10.1051/parasite/2013049

**Published:** 2013-12-09

**Authors:** Roberto Daibes Naiff, Toby Vincent Barrett

**Affiliations:** 1 Coordenação Sociedade, Ambiente e Saúde, Instituto Nacional de Pesquisas da Amazônia – INPA Av. André Araújo, 2.936, Petrópolis CEP 69011-970 Caixa Postal 478 Manaus AM Brasil

**Keywords:** *Trypanosoma lainsoni* n. sp., trypomastigotes, *Mesomys hispidu*s, Rodentia, Caviomorpha, Brazil

## Abstract

We report the detection, isolation and description of *Trypanosoma* (*Megatrypanum*) *lainsoni* n. sp. from a caviomorph rodent, *Mesomys hispidus* (Rodentia: Echimyidae), obtained in the Rio Negro region of the state of Amazonas, in northern Brazil. Laboratory-bred white mice (*Mus musculus*) and rats (*Rattus rattus*) were inoculated with large numbers of culture forms by intraperitoneal route, and trypomastigotes appeared in their blood 3–8 days post-inoculation. One single epimastigote was also found in *Mus musculus*. Similar attempts to infect *Rattus norvegicus*, hamsters (*Mesocricetus auratus*), the opossum *Didelphis marsupialis*, the anteater *Tamandua tetradactyla* and triatomine bugs were unsuccessful, following six months of observations and microscopic examinations of blood films and blood cultures. As we have found no previous record of a *Trypanosoma (Megatrypanum)* species naturally infecting a member of the family Echimyidae, or any other caviomorph rodent, we conclude that this is the first time such an infection has been reported. The new species is unusual in the subgenus for its infectivity to laboratory mice.

## Abstract (fr)

Nous rapportons la détection, l’isolement et la description de *Trypanosoma* (*Megatrypanum*) *lainsoni* n. sp. d’un rongeur caviomorphe, *Mesomys hispidus* (Rodentia: Echimyidae) de la région de Rio Negro dans l’État d’Amazonas, au nord du Brésil. Des souris blanches de laboratoire (*Mus musculus*) et des rats (*Rattus rattus*) ont été inoculés avec de nombreuses formes en culture par voie intrapéritonéale, et des trypomastigotes sont apparus dans leur sang 3–8 jours après inoculation. Un seul épimastigote a aussi été trouvé chez *Mus musculus*. Des tentatives similaires pour infecter *Rattus norvegicus*, des hamsters (*Mesocricetus auratus*), l’opossum *Didelphis marsupialis*, le fourmilier *Tamandua tetradactyla* et des punaises Triatominae ont été infructueuses après six mois d’observations et d’examens microscopiques de films sanguins et de cultures de sang. Nous n’avons trouvé aucune mention préalable d’une espèce de *Trypanosoma (Megatrypanum)* infectant naturellement un membre de la famille Echimyidae, ou n’importe quel autre rongeur caviomorphe, et nous concluons donc que c’est la première fois qu’une telle infection est rapportée. La nouvelle espèce est particulière dans le genre par son infectiosité pour la souris de laboratoire.

## Introduction

In the last few decades, the number of recorded species of trypanosomes has increased considerably and the medical and veterinary importance of these parasites has been discussed in many textbooks of protozoology, parasitology and related subjects.

The subgenus *Megatrypanum* is a somewhat heterogeneous group of large trypanosomes. The internal structure of members of this genus is characterised by the kinetoplast which is typically placed far from the posterior end of the parasite and near the nucleus [13]. When blood forms can be found in stained blood films, two morphologically different trypomastigotes can often be seen; one is large, but slender and the other large and broad. Frequently, however, the infection in the natural host can only be detected following haemoculture. All species of *Megatrypanum* are normally non-pathogenic in their natural hosts [6].

Data regarding reproduction in the mammalian host, vectors and life cycles of members of the subgenus *Megatrypanum* are fragmentary or completely lacking for most species, and many of these trypanosomes appear to be restricted to individual species or genera of their mammalian hosts [5, 11]. The great majority of these trypanosomes are strictly host-specific in their natural environment [6].

The species described here is the first species of the subgenus *Megatrypanum* [5] to be described in a caviomorph rodent. It was isolated, in NNN culture medium, from a spiny tree-rat obtained in the mid-Rio Negro region of Amazonas State, northern Brazil.

## Material and methods

An adult male specimen of the spiny tree-rat, *Mesomys hispidus*, was killed, by a collector of “piassaba”, in the crown of the palm tree *Leopoldinia piassaba* Wallace, in lowland rainforest near the Igarapé Japaumerí, a stream flowing into the River Padauarí, northern Amazonas State, Brazil. The rat was necropsied under field conditions after asepsis with iodated alcohol, and heart blood then inoculated into seven tubes of diphasic NNN blood-agar culture medium [10]; two thin blood films were fixed in absolute methyl alcohol and stained by Giemsa’s method [2]. Fragments of skin tissue from the nose, ears and base of the tail were triturated in saline solution (0.9% NaCl) containing penicillin (200 U/mL) and streptomycin (0.312 mg), incubated at room temperature for 2 h and then used to inoculate three-month-old hamsters. Two hamsters were inoculated intradermally with the suspension into the nose and rear paws, and a similar suspension of triturated liver and spleen was inoculated intradermally and intraperitoneally into two other hamsters. Following successful isolation of a trypanosome in the NNN culture medium (first sub-passage), the culture forms were inoculated into 21-day-old laboratory mice by the intraperitoneal route. Measurements of the trypomastigotes (Table 1) follow Hoare [6]. All measurements are in μm.

**Table 1.** *Trypanosoma (Megatrypanum) lainsoni* n. sp. Linear measurements (μm) and morphometric indices of trypomastigote forms in mouse peripheral blood. Smears stained by Giemsa’s method.

Parameters	Minimum value	Maximum value	Mean	*SD*
L	28 (Figure 2)	37 (Figures 3, 4, 20)	33.4	2.523
F	4 (Figure 2)	10 (Figures 3, 4, 20)	7.7	1.371
NA	10 (not shown)	16 (Figure 8)	13.8	1.490
PN	10 (Figures 2, 6, 9)	14.5 (Figure 13)	12.4	1.379
PK	6 (not shown)	11 (Figure 11)	8.2	1.079
KN	2 (Figures 2, 10, 12)	6 (Figure 19)	4.1	1.130
W	3 (Figure 16)	8 (Figure 6)	4.5	0.870
NI	0.67 (Figure 9)	1.36 (Figure 7)	0.93	1.140
KI	2.2 (not shown)	6 (Figures 10, 12)	3.29	0.992
W/L	0.09 (Figure 16)	0.24 (Figure 6)	0.14	0.033
F/L	0.14 (Figure 2)	0.28 (Figure 15)	0.23	0.036

Sample size = 28. *SD* = standard deviation. L = total length including free flagellum. F = length of the free flagellum. NA = distance from the middle of nucleus to the anterior end. PN = middle of the nucleus to the posterior end. PK = posterior end to the kinetoplast. KN = middle of nucleus to the kinetoplast. W = width. NI = nuclear index (PN/NA). KI = kinetoplastic index (PN/KN). Terminology according to Hoare (1972).

## *Trypanosoma* (*Megatrypanum*) *lainsoni* n. sp.

urn:lsid:zoobank.org:act:7DA83760-8EA2-44D3-BBC4-8C9864F948A6

Type host: *Mesomys hispidus* (Desmarest, 1817) (Rodentia: Echimyidae).

Type locality: municipality of Barcelos (00°S 64°W), state of Amazonas, Brazil.

Collector and date: Francisco Lima Santos, July 17, 1995.

Material examined: Hapantotypes, Giemsa-stained films of peripheral blood of experimentally infected laboratory mice: one epimastigote and 28 trypomastigotes, deposited in the Muséum National d′Histoire Naturelle, Paris, France (MNHN) under registration number MNHN ZS126.

Vector: Unknown.

Strain-code: IM-4156 (Laboratory designation).

Etymology: Specific name in recognition of Professor Ralph Lainson’s contributions to the study of protozoan parasites in the Amazonian fauna of Brazil.

### Description (Figures 1–20; Table 1)

Bloodstream trypomastigotes (Figures 2–20) with a mean length of 33.4 including free flagellum and width 4–6. Free flagellum 4.0–10.0 (mean 7.7); undulating membrane well developed. Posterior end of the body long and pointed (Figures 17–20) or cuneiform (Figure 4); anterior end tapering to free flagellum. Nucleus oval, longitudinal or transverse near middle of body with a nuclear index (NI) of 0.7–1.4, but mainly 0.9–1.1; kinetoplast closer to nucleus than to posterior end with kinetoplastic index (KI) of 2.2–6.0, but mainly 0.9–1.1, and marginal. Other measurements are given in Table 1. A single bloodstream epimastigote was detected, with its nucleus in a strongly posterior position and NI of 0.4 (Figure 1).

**Figures 1–20.**
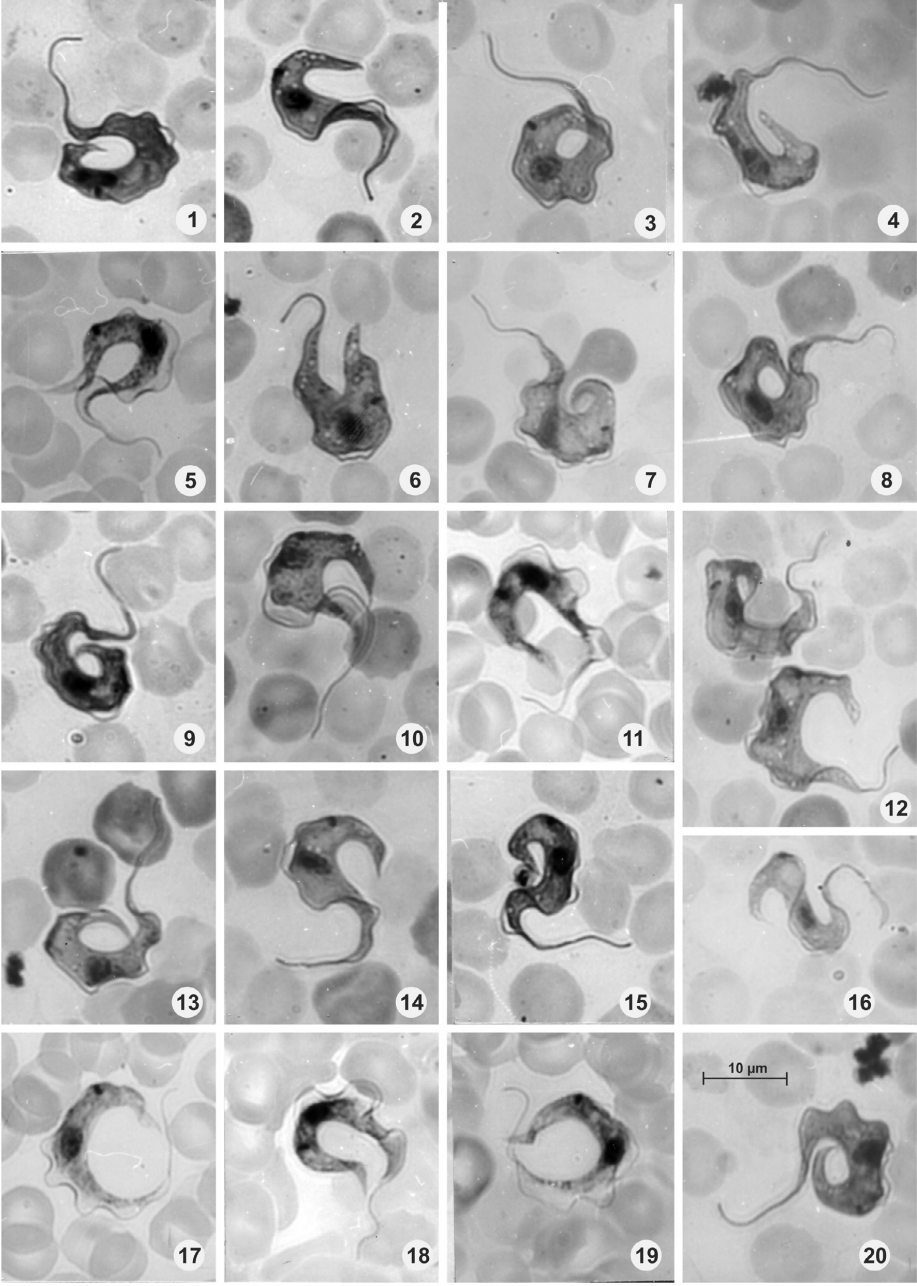
*Trypanosoma (Megatrypanum) lainsoni* n. sp., parasites found in peripheral blood of experimentally infected mice; Figure 1: Epimastigote with signs of nuclear division; Figures 2–20: Trypomastigotes. Scale in Figure 20.

### Animal inoculation and culture

Following periodic examination over a period of six months none of the hamsters inoculated with tissues of *Mesomys hispidus* showed any evidence of infection with *Leishmania* at the sites of inoculation, or the presence of trypanosomes in their blood. This clearly indicated the absence of *Leishmania* infection in *M. hispidus* and that hamsters could not be infected by *T. (M.) lainsoni* n. sp.

One of the seven original cultures of the spiny rat’s blood produced good epimastigote growth of a trypanosome; four tubes were contaminated by bacteria and fungi and although two tubes escaped such contamination they had not isolated the trypanosome. Three to eight days post-inoculation (p.i.) of the culture forms, mice (*Mus musculus*) showed an average of 5–6 trypomastigotes per microscope field (magnification × 40) in fresh preparations, and at five months p.i. parasitaemia was sub-patent microscopically but still demonstrable in some mice by haemoculture. One single epimastigote was also found in mice.

Inoculation of other animals with flagellates of 31-day-old culture forms gave the following results after microscopic examination of fresh blood preparations or haemoculture. One 30-day-old *Rattus rattus* showed 1–3 trypomastigotes per field on day 3 p.i. By haemoculture two 16-day-old *Rattus norvegicus* were negative, as were one juvenile *Tamandua tetradactyla* and two young *Didelphis marsupialis.*

### Xenodiagnosis using triatomine bugs

Twenty *Rhodnius pictipes,* 20 *R. robustus* and 20 *R. brethesi* were fed on mice showing abundant trypomastigotes in their peripheral blood and examined after 3–6 weeks. All failed to become infected.

## Discussion

We have been unable to find any previous record of a trypanosome of the subgenus *Megatrypanum* in echimyid rodents or any other caviomorphs (The Zoological Record 1978 to 2013, Scopus, PubMed, ISI).

Species of the subgenus recorded in neotropical rodents include *T*. *phyllotis* [4], from *Phyllotis* spp (Cricetidae) in semi-arid regions of western Peru; *T*. *amileari* [7], from *Oligoryzomys eliurus* [11] (Cricetidae) in Brazil (northern Goiás; now the State of Tocantins), *T*. *rochasilvai* [10] from *Oryzomys* sp. (Cricetidae) in the State of São Paulo, Brazil; and *T*. *zeledoni* [3], from *Liomys salvini* (Heteromyidae). With the exception of *T*. *phyllotis*, attempts to cultivate these trypanosomes in NNN blood-agar medium or to infect mice and rats have been unsuccessful and, apart from *T*. *amilcari* (*L* = 32–40) all are, on average, much larger than *T*. *lainsoni. T*. *phyllotis* was cultured in NNN medium and infected the sand fly *Lutzomyia noguchi* and laboratory rats of up to six days old. The mean length of *T. phyllotis* was given as 47, while that of *T*. *lainsoni* was only 33.4; the kinetoplast of the former was smaller than that of the latter and the nucleus more rounded. The kinetoplast of *T. amileari* was shown to be closer to the nucleus (KN 2–6, KI 5) than that of *T*. *lainsoni*; *T*. *rochasilvai* was very much larger (*L* = 50–73 vs. 28–37). The length of *T*. *zeledoni* was given as 36–56; the nucleus is placed more strongly anterior than that of *T*. *lainsoni* (NI = 1.4–1.5 vs. 0.67–1.36) and the kinetoplast is rounded and sub-marginal rather than marginal: finally, the flagellum is relatively short compared with that of *T*. *lainsoni.*

As is the case for many other members of the subgenus *Megatrypanum*, the above criteria for separating species may seem to be somewhat tenuous in the absence of direct comparison under controlled conditions [12]. Species of this subgenus, however, are in general host-restricted [5], and the fact that our isolate is from a distinct mammalian suborder (Caviomorpha) has influenced our decision to accord its specific status. We also feel that it is useful to make a name available for a parasite which is of potential use as a new trypanosome laboratory model.

Our failure to experimentally infect three species of phlebotomine sand flies does not necessarily preclude *Lutzomyia* species as vectors of *T. (M.) lainsoni*. Apart from *T*. *phyllotis*, other mammalian *T*. (*Megatrypanum*) species suspected to have a phlebotomine vector are *T*. *leonidasdeanei* [14], probably transmitted by *Lutzomyia vespertilionis*, and *T*. *freitasi* [9], found in a wild-caught *Lutzomyia claustrei* [8].

Epimastigotes thought to belong to a *T*. (*Megatrypanum*) species were observed in lymphoid tissues of the armadillo *Dasypus novemcinctus* [1], but our Figure 1 of *T*. *lainsoni* is probably the first record of the epimastigote stage of a *T. (Megatrypanum)* species, other than that of *T*. *theileri,* in mammalian blood. The elongated form of the nucleus and its pattern of staining are suggestive of an early stage of division, and the structure just anterior to the nucleus looks like a small, second kinetoplast and associated flagellum.

The potential of Aristides Herrer’s *Phyllotis*/*Lutzomyia noguchii* model appears to have been neglected by later students of *Megatrypanum*, and we suggest that the behaviour of *T*. *lainsoni* in mice could provide a rewarding area of study of these elegant and phylogenetically ancient trypanosomes.
